# Implantation of the Bonebridge BCI 602 after Mastoid Obliteration with S53P4 Bioactive Glass: A Safe Method of Treating Difficult Anatomical Conditions-Preliminary Results

**DOI:** 10.3390/life11050374

**Published:** 2021-04-22

**Authors:** Bartłomiej Król, Katarzyna Beata Cywka, Magdalena Beata Skarżyńska, Piotr Henryk Skarżyński

**Affiliations:** 1 World Hearing Center, Institute of Physiology and Pathology of Hearing, 02-042 Warsaw, Poland; b.krol@ifps.org.pl (B.K.); k.cywka@ifps.org.pl (K.B.C.); m.skarzynska@csim.pl (M.B.S.); 2 Institute of Sensory Organs, 05-830 Kajetany, Poland; 3 Center of Hearing and Speech Medincus, 05-830 Kajetany, Poland; 4 Heart Failure and Cardiac Rehabilitation Department, Second Faculty of Medicine, Medical University of Warsaw, 03-242 Warsaw, Poland

**Keywords:** S53P4 bioactive glass, cholesteatoma, mastoid obliteration, otosurgery, Bonebridge BCI 602, bone conduction implant

## Abstract

This study presents the preliminary results of a new otosurgical method in patients after canal wall down (CWD) surgery; it involves the implantation of the Bonebridge BCI 602 implant after obliteration of the mastoid cavity with S53P4 bioactive glass. The study involved eight adult patients who had a history of chronic otitis media with cholesteatoma in one or both ears and who had had prior radical surgery. The mean follow-up period was 12 months, with routine follow-up visits according to the schedule. The analysis had two aspects: a surgical aspect in terms of healing, development of bacterial flora, the impact on the inner ear or labyrinth, recurrence of cholesteatoma, and possible postoperative complications (firstly, after obliteration of the mastoid cavity with S53P4 bioactive glass, then after implantation). The second was an audiological aspect which assessed audiometric results and the patient’s satisfaction based on questionnaires. During the follow-up period, we did not notice any serious postoperative complications. Studies demonstrated significantly improved hearing thresholds and speech recognition in quiet and noise using the Bonebridge BCI 602. Data collected after six months of use showed improved audiological thresholds and patient satisfaction. Based on the preliminary results, we believe that the proposed two-stage surgical method using bioactive glass S53P4 is a safe and effective way of implanting the Bonebridge BCI 602 in difficult anatomical conditions. This makes it possible to treat a larger group of patients with the device.

## 1. Introduction

Patients with chronic otitis media with cholesteatoma are often treated with surgery: preserving of the posterior wall of the ear canal (also called canal wall up, CWU), and in advanced stages of the disease, radical mastoidectomy (canal wall down, CWD) [1,2,3]. Despite the appreciably smaller number of recurrences of cholesteatoma with the CWD technique [4], epidermis and cerumen accumulate in the postoperative cavity [5], which requires frequent follow-up visits. The postoperative cavity must be protected from water, and some patients complain of dizziness due to wind or water [6,7]. Ear inflammation recurs more often, and surgical revisions are necessary if antibiotics are ineffective [8].

The above problems have an impact on the patient’s quality of life, so for over 100 years, the problem of obliteration of the mastoid cavity has been a challenge for generations of otosurgeons [9]. Long-term results with autologous material—bone shavings, cartilage, fat, muscle flaps, and fascia—have not been satisfactory [10,11,12,13], nor have hydroxyapatite, ceramics, and cement [14,15]. Currently, some years of experience indicate that bioactive glass (S53P4) is a proper for reconstructing the posterior wall of the ear canal and obliterating the postoperative cavity [16,17].

In everyday clinical practice, we often (from 10 to even 40% of cases) [18] meet patients who have leakages from their ears when trying to fit a classic hearing aid [8,19]; such patients are qualified for a Bonebridge bone-conduction hearing implant. However, after further diagnostics involving CT of the temporal bone, patients have been repeatedly disqualified from the Bonebridge device due to a lack of suitable anatomical conditions for safe implantation; our center prefers not to implant the device outside the mastoid process, although in some centers this technique is used [20,21,22].

In this paper, we propose a combination of two surgical techniques that will enable the use of the Bonebridge BCI 602 implant in a wider group of patients [23,24].

## 2. Materials and Methods

For many months, our clinic has been carrying out mastoid obliteration procedures with S53P4 bioactive glass, and eight adult patients were selected from this group. In our clinic, from October 2016 to January 2020, we have performed over 100 mastoid obliteration procedures using bioactive S53P4 glass. From this group, patients who had limitations in wearing classic hearing aids after reconstruction of the posterior wall of the external auditory canal were selected. Eight patients gave their written consent to participate in the project. The selected group consists of patients who, after examination with a conventional hearing aid, complained about leakage from the ear (both before and after obliteration of the mastoid cavity with bioactive glass) and were qualified for bone conduction device implantation after the diagnostic procedure. They were five women and three men, aged 25 to 71, mean age 49.3 years, who had a history of chronic otitis media in one or both ears and who had had CWD mastoidectomy. No ear leakage had been observed in the study group for at least 12 months. CT examination of the temporal bones revealed no anatomical positions for implantation of the Bonebridge BCI 601 device. All patients had stable hearing thresholds and did not report dizziness or other vestibular disorders. The patient’s medical history was analyzed, as well as other diseases that might affect the healing process. Patients said they had no allergies to drugs or chemicals.

During the preoperative micro-otoscopic examination, a dry cavity was found after radical surgery in each case, and swabs were taken (results in Table 1). During the qualification process, we selected only healthy ears in the otoscopic examination; others with otitis were disqualified and treated accordingly. Pure tone audiometry was performed at octave frequencies (0.25–8.0 kHz). The next day, the mastoid cavity was obliterated.

Follow-up included routine visits after seven days, then one, three, and six months after mastoid obliteration with S53P4 bioactive glass surgery. After seven days, the dressing was removed from the ear, as were the sutures from the retroauricular incision; no complications were found. Some 30 days after surgery, the area was cleaned, a control swab was taken, and pure tone audiometry conducted. Microbiological testing indicated no growth of bacteria or fungi in all cases. At subsequent visits three and six months later, otoscopy showed correct healing. Air and bone conduction thresholds (at octave frequencies 0.25–8.0 kHz) showed results comparable to those before surgery. At six months after the procedure, a HRCT scan of the temporal bones was made: the image showed a correctly healed Bonalive reconstruction, with no evidence of bone destruction and sufficient conditions for Bonebridge implantation in each case, making it possible to proceed to the next stage of treatment.

For all the group members, we simulated the result of using a bone conduction implant based on a sound processor (Baha 4) adjusted to a bone conductive device on a softband. Audiometric tests were performed before implantation (pure tone audiometry, free field audiometry, and speech audiometry in quiet and noise), and these showed a potential significant improvement in hearing and for understanding speech. Free-field audiometry was performed in an adapted soundproof room. Word audiometry in free-field was performed under the same conditions as threshold audiometry: measurements were performed in silence with speech levels of 70 dB SPL using monosyllabic words from a list of Polish words. The speech reception threshold in noise was assessed using the Polish matrix sentence test [25]. The noise level was fixed at 65 dB SPL. These tests confirmed that all the patients met the audiological criteria qualifying them for using a Bonebridge BCI 602 [26].

The Bonebridge device was implanted six to seven months after obliteration. The dressings were removed as well as sutures from the retroauricular incision seven days after surgery. After 30 days, an otoscopic examination showed normal healing; a CT scan of the temporal bone was performed. An audiometric examination (at octave frequencies 0.25–8.0 kHz) showed no deterioration of hearing. The device was set up and the settings adjusted to the audiometric test results and the patient’s subjective assessment.

At one and six months after implantation, we compared bone conduction thresholds at 500, 1000, 2000, and 4000 Hz and the percentage of correct answers in speech audiometry in quiet and noise at 65 dB with and without the implant. The APHAB questionnaire (abbreviated profile of hearing aid benefit) was used before and after implantation to assess the benefits in everyday conditions.

All procedures were performed in the years 2019 to 2020 under general anesthesia according to the schemes described in previous publications [23,27], by one otosurgeon with many years of experience. The prospective study was approved by the Bioethical Committee of the Institute of Physiology and Pathology of Hearing, Warsaw, Poland (nr KB/17/2016). All patients gave written consent to participate in the project.

### Statistical Analysis

The assumption of normal distribution of the variables was tested with a Shapiro–Wilk test. The majority of variables were normally distributed, so a paired *t*-test or a repeated measures ANOVA were used to compare the outcomes before and after implantation. The level of statistical significance was set at *p* < 0.05. All statistical tests were performed using IBM SPSS Statistics v.24.

## 3. Results

The surgical procedures were uneventful, and there was no vomiting or increased body temperature. Pain was not bothersome and subsided after non-narcotic analgesics (after seven days, pain was reported by three patients). Seven days after surgery, internal dressings and external sutures were removed, without serious complications, and no purulent discharge or bleeding was observed. The external auditory canal was reconstructed with the correct shape, and what is more, there was a significant reduction in the dimensions of the postoperative cavities. The postauricular area was in all cases without erythematous edema or other symptoms of infection. Patients reported no dizziness, nausea, or loss of hearing.

Despite the correct otoscopic image before the obliteration of the cavity and the negative history of ear leakage a few months previously, bacterial colonization was still present; in two cases, there was colonization by *Staphylococcus aureus*, and in another, *Pseudomonas aeruginosa*. It is worth mentioning, however, that all postoperative swabs were negative, although in four cases, watery leakage was observed, and one patient reported pain. In case of edema or leakage, fluocinolone acetonide ointment was applied to the posterior wall of the ear canal. Only one patient reported mild pain.

**Figure 1:**
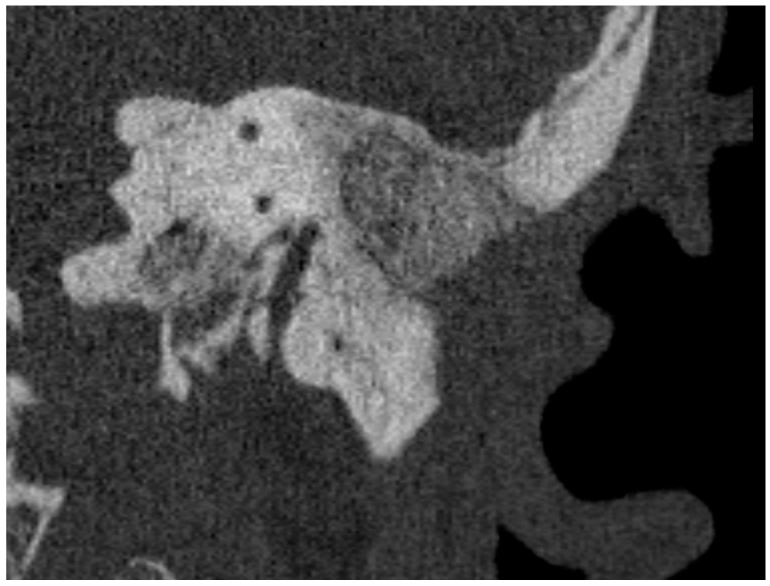
Cross-section of the left temporal bone in the sagittal plane with the area filled with bioactive glass.

Micro- and videotoscopic examination three months after the surgery showed a healed reconstruction of the posterior wall of the ear canal in all patients, with no signs of leakage of granulation tissue and no pain. After six months, otoscopic examination showed no signs of pathology, and control swabs from smears of the operated ear were negative (Table 1). A control high-resolution CT was performed which showed complete healing of the surgical site, and no bone destruction was found, suggesting no recurrence of cholesteatoma (Figure 1).

Healing of the successful mastoid reconstruction enabled the Bonebridge implant procedure to be performed. Based on the experience and awareness of the operator and the initial analysis of CT images before the reconstruction of the filling with bioactive glass S53P4, it was possible to safely place the Bonebridge implant in each of the studied cases without the use of BCI lifts. Our observations show that when drilling in bioactive glass, special care needs to be taken because in all cases the S53P4 bioactive glass was harder than bone and it is not easy to correctly prepare the site for implantation.

In all cases, implantation was performed without edema or hematoma over the implant or other complications, first at the observation period seven days later, then after four to five weeks and at six months. A CT performed six months after implantation showed the FMT transducer partially in the bone and partially in the S53P4 bioactive glass reconstruction, with safe bone margins around the external auditory canal, dura, and sigmoid sinus.

The existence of comorbidities (degeneration of the spine, hypertension, peptic ulcer, hypothyroidism, benign prostatic hyperplasia) did not affect healing.

Comparing the results before surgery and after the first and second surgeries, the results of air conduction (AC), bone conduction (BC), and the air-bone gap showed stable hearing thresholds at octave frequencies of 0.25 to 8.0 kHz (Table 2).

### 3.1. Audiological Results

#### 3.1.1. Participants

The study was carried out on eight adult participants: five females and three males aged 26 to 74 years (mean, 50.5). Five patients were implanted on the left ear, and three on the right.

To assess the auditory benefits from the Bonebridge, audiometric tests were performed: pure tone audiometry at octave frequencies of 0.25 to 8.0 kHz, free-field audiometry, and speech understanding in quiet and noise (matrix sentence test). To assess the auditory benefits of using the implant, the patient completed an APHAB questionnaire (abbreviated profile of hearing aid benefit).

#### 3.1.2. Bone Conduction

Bone conduction remained stable after surgery, and in all patients, the threshold shift was less than ± 10 dB. The mean bone conduction threshold before surgery was M = 19.06 (SD = 7.64); after surgery, it was M = 18.19 (SD = 7.52); the difference was statistically nonsignificant, *t* = 1.02; *p* = 0.341. There were no significant differences of BC thresholds before and after surgery at any of the tested frequencies (BC 500 Hz, *t* = 1.18; *p* = 0.275; BC 1000 Hz, *t* = 0.01; *p* > 0.999; BC 2000 Hz, *t* = 1.53; *p* = 0.170; BC 4000 Hz, *t* = 0.76; *p* = 0.470).

#### 3.1.3. Free-Field Audiometry Outcomes

**Figure 2:**
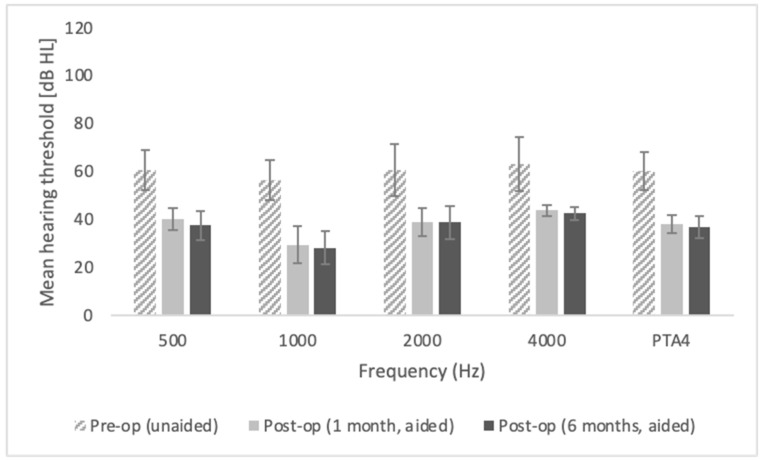
Hearing thresholds obtained in free-field audiometry.

The mean hearing threshold obtained in free-field audiometry before surgery (unaided) was 60.2 dB HL (SD = 7.78); at one month post-op (aided) it was 38.0 dB HL (SD = 3.7), and at six months post-op (aided) it was 36.7 dB HL (SD = 4.5); the improvement was statistically significant, *F* = 167.66; *p* < 0.001; *e*^2^ = 0.969. The mean functional gain observed at the six-month follow-up was 23.4 dB. The functional gain was similar across all frequencies: for 500 Hz, it was 23.1 dB; 1000 Hz, 28.1 dB; 2000 Hz, 21.9 dB; and 4000 Hz, 20.6 dB. Detailed outcomes of free-field audiometry are shown in Figure 2.

All the differences between pre-op and both post-op follow-ups are statistically significant at *p* < 0.001; all the differences between post-ops (one month and six months) are statistically non-significant. The bars represent mean scores, the error bars represent standard deviations.

#### 3.1.4. Free-Field Word Recognition in Quiet

**Figure 3:**
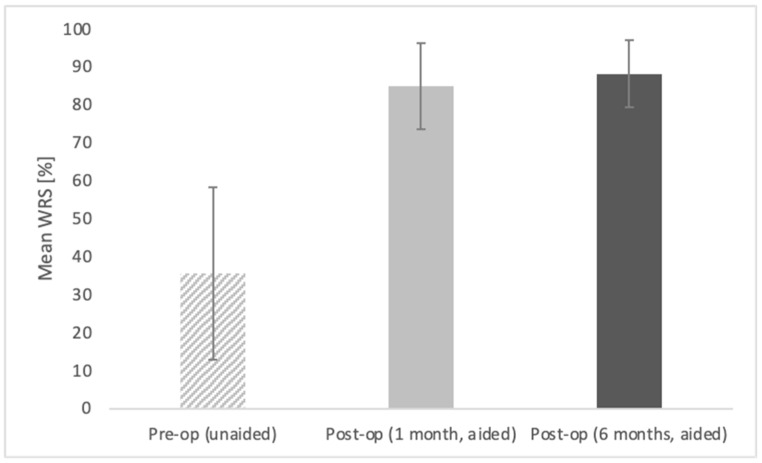
Word recognition scores (WRS) in quiet obtained in free-field audiometry at 70 dB SPL.

An improvement in speech recognition in quiet was observed (Figure 3). The mean word recognition score obtained in free-field audiometry before surgery (unaided) was 35.6% (SD = 22.8), at one month post-op (aided) it was 85 % (SD = 11.4), and at six months post-op (aided) it was 88.1 % (SD = 8.8). The improvement was statistically significant (*F* = 33.62; *p* < 0.001; *e*^2^ = 0.828).

The differences between pre-op and both post-ops are statistically significant at *p* < 0.001; the difference between post-ops (one month and six months) are statistically non-significant. The bars represent mean scores, the error bars represent standard deviations.

#### 3.1.5. Intelligibility of Speech in Noise

**Figure 4:**
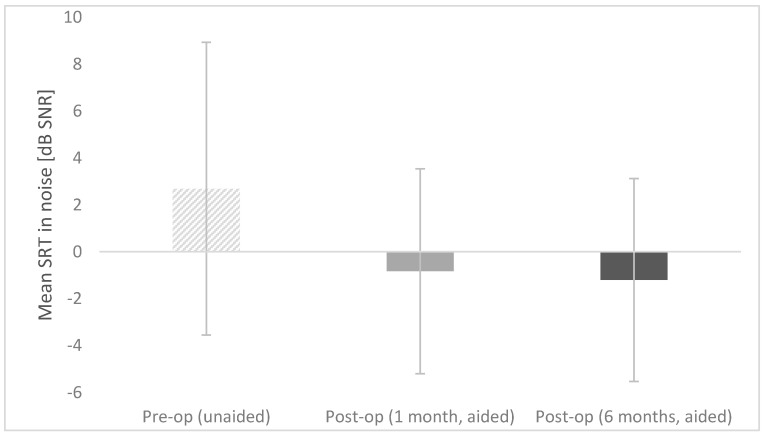
Preoperative and postoperative speech reception threshold (SRT) in noise.

The speech reception threshold in noise improved significantly after implantation in comparison with that before implantation (*F* = 13.06; *p* = 0.008; *e*^2^ = 0.651). Before surgery, it was M = 2.69 dB SNR (SD = 6.24); at one month post-op M = −0.83 dB SNR (SD = 4.37); at six months post-op M = −1.20 dB SNR (SD = 4.33). Figure 4 shows the mean speech reception thresholds in noise obtained preoperatively (unaided) and postoperatively (aided).

The differences between pre-op and both post-ops are statistically significant at *p* < 0.001; the difference between post-ops (one month and six months) are statistically non-significant. The bars represent mean scores, the error bars represent standard deviations.

### 3.2. Results of the APHAB Questionnaire

**Figure 5:**
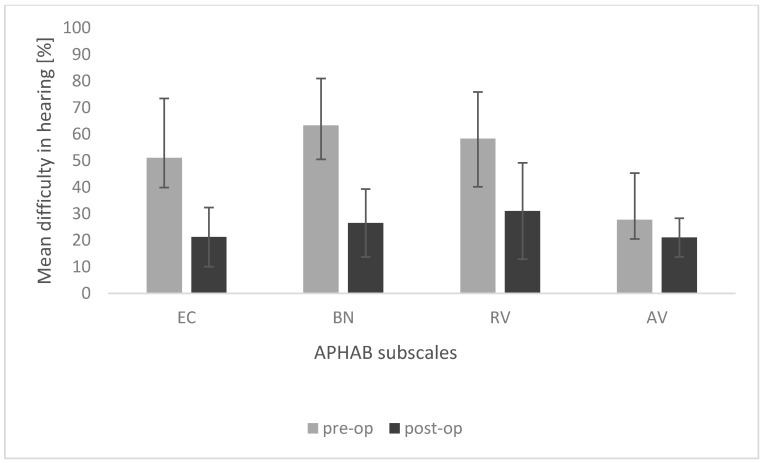
Preoperative and postoperative results of APHAB questionnaire.

The results of the APHAB questionnaire obtained before and after implantation showed a statistically significant improvement (reduction of problems) on the first three subscales. In the subscale Ease of Communication, the mean score before surgery was M = 51.02; SD = 22.43, and significantly improved after surgery to M = 21.22; SD = 11.14; *t* = 4.46; *p* = 0.003. Additionally, on the subscale Background Noise, the mean score significantly improved from M = 63.31; SD = 17.62 before surgery to M = 26.48; SD = 12.78 after surgery; *t* = 5.55; *p* = 0.001. The same was true for improvement on the Reverberation subscale, from M = 58.33; SD = 17.53 before surgery to M = 31.04; SD = 18.17 after surgery; *t* = 5.28; *p* = 0.001. The only exception was the mean score on the Aversiveness subscale, which was similar before surgery (M = 27.73; SD = 17.63) to that obtained after surgery (M = 21.02; SD = 7.26; *t* = 1.35; *p* = 0.221). Figure 5 shows the mean APHAB scores obtained before and after implantation.

EC, ease of communication; BN, background noise; RV, reverberation; AV, aversiveness. The differences in EC, BN, and RV subscales are statistically significant at *p* < 0.05; the difference in the AV subscale is statistically non-significant. The bars represent mean scores, the error bars represent standard deviations.

## 4. Discussion

Beginning with publications by Mosher et al. [9], the technique of obliteration of the mastoid cavity has become an increasingly popular method of treatment. Nevertheless, each author’s technique differs [28].

Over the years, various attempts have been made to use bone shavings, cartilage, fat, muscle flaps, and fascia [10,11,12,13]; however, the long-term effects have not been satisfactory due to a tendency to shrink and resorb. Moreover, using this method, it is possible to accidentally introduce cholesteatoma. Subsequent attempts with hydroxyapatite, ceramics, and cement have not brought long-term and positive effects [14,15].

Recent publications focus on bioactive glass (S53P4) as an efficient material for reconstructing the posterior wall of the ear canal and obliterating the postoperative cavity [14,29,30]. This material is widely used in dentistry and maxillofacial surgery, as well as in orthopedics (benign bone tumors, injuries) and neurosurgery. Safety is confirmed by its receipt of an EU Declaration of Conformity (CE) in 2004, and registration by the U.S. FDA in 2008. Its specific composition (53% SiO_2_, 23% Na_2_O, 20% CaO, 4% P_2_O_5_) has osteostimulating properties, preventing the development of cholesteatoma, and provides a constant volume for a long time. Its antibacterial action against pathogens (*Streptococcus pneumonia*, *Staphylococcus aureus*, *Pseudomonas aeruginosa*, *Moraxella catarralis*, *Haemophilus*) [31,32] has been proven many times, and also in the case of contact with implantable devices [33]. An important feature of bioactive glass S53P4 is that it is safe to use in direct contact with the sigmoid sinus [28] or dura [29] without complications when using topical cartilage or fascia [34].

In the present study, healing was normal in all cases. The presence of a watery discharge in four cases (50%) cannot be treated as a pathology, since the results of swabs from this period showed negative outcomes in all cases compared to some positive results before surgery. Similar to previous reports [14,15] it confirms the antibacterial properties of bioactive glass. Referring to other studies on similar topics [16,28,35,36], healing in the initial period was complicated even in 30% of cases (local infections) with a positive result of treatment with local or systemic antibiotics. Therefore, expecting long-term results of the procedure in the case of mastoid obliteration is at this stage premature. Some authors even recommend, empirically, the use of antibiotic drops in combination with antifungal drops in the first days after dressing removal until the results of smears are obtained [27].

During the observation period, no dizziness or hearing impairment was observed, which, in combination with the postoperative HRCT evaluation, demonstrates the absence of any toxic effect of S53P4 bioactive glass on the cochlea [17,37] and on the structures of the labyrinth [38].

HRCT performed six months after obliteration showed no recurrence of cholesteatoma or displacement of the bioactive S53P4 in any of the cases; we did not notice any inflammatory reaction [17,28,36], bone erosion, or destruction, so there was no suspicion of recurrence of cholesteatoma. We therefore departed from the presumed necessity [39] to perform an MRI in these patients. In our clinic, performing an HRCT after reconstruction is necessary due to the need to evaluate the healing of the bioactive glass and to assess the anatomical conditions for the Bonebridge FMT. In case of doubt, the patient is immediately referred for a comparative MRI examination, which has greater sensitivity [39].

In all patients implanted with the Bonebridge BCI 602, we observed improved hearing and better speech understanding in both quiet and noisy environments. The patient’s subjective opinion is an essential element of the assessment, and here, we used the APHAB questionnaire [40]. The APHAB consists of 24 questions divided into four subscales that measure the impact of hearing loss in everyday listening situations [41]. Our patients’ results showed that hearing quality of life improved significantly after implantation and remained stable for at least six months.

Since some authors perform obliteration of the mastoid with bioactive glass simultaneously with the CWD surgery, one may ask whether the procedure of simultaneous obliteration of the mastoid cavity and implantation is possible. Theoretically it is; however, it is not performed in our clinic as we cannot guarantee that we will obtain a sufficiently large and stable implant bed, which is, after all, one of the crucial elements of our method and determines the possibility of safe implant placement in a typical place. In case of any complications resulting from the use of the bioactive glass, there is a risk of implant damage. The only indication for Bonebridge implantation with obliteration is the patient’s general health burden.

## 5. Conclusions

Preliminary studies have shown the safety and effectiveness of the two-stage surgical procedure involving obliteration of the cavity from CWD mastoidectomy with the use of bioactive glass S53P4 followed by implantation of the Bonebridge BCI 602. The result of the operation is a closed cavity after radical surgery and a safely placed bone conduction device that improves hearing, a result that significantly improves the patient’s quality of life. We plan to expand the research and wish to popularize the method.

## Figures and Tables

**Table 1:** Results of swabs from patients.

Gender	Age	Previous Surgeries (Number)	Result of Ear Swab before Surgery	Healing at One Month	Result of Ear Swab Six Months after Surgery
F	66	2	*Staphylococcus aureus* (medium growth ++)	Clear leakage, pain	Negative
F	53	2	Negative	Proper, dry ear	Negative
M	24	5	Negative	Clear leakage	Negative
F	60	4	*Staphylococcus aureus* (abundant growth +++)	Proper, dry ear	Negative
M	74	3	Negative	Proper, dry ear	Negative
M	55	2	Negative	Proper, dry ear	Negative
F	31	3	*Pseudomonas aeruginosa* (medium growth ++)	Clear leakage	Negative
F	31	4	Negative	Clear leakage	Negative

**Table 2:** Mean values of air conduction (AC), bone conduction (BC), and air-bone gap (ABG) before and after surgery.

	AC (dB)	BC (dB)	ABG (dB)
Before surgery	63.2	24.6	38.6
Six months after surgery	63.3	27.2	36.1

## Data Availability

The datasets used and/or analysed during the current study are available from the corresponding author on reasonable request.
